# The Geriatric Nutritional Risk Index and its association with all-cause mortality in cancer patients with sepsis: a dual-center retrospective cohort study

**DOI:** 10.3389/fnut.2026.1795795

**Published:** 2026-07-14

**Authors:** Yili He, Jie Zhang, Xiaojin Yuan, Yang Li, Wenyan Jiang, Ling Huang

**Affiliations:** Department of Critical Care Medicine, Guangxi Medical University Cancer Hospital, Nanning, China

**Keywords:** cancer, Geriatric Nutritional Risk Index, mortality, predictive value, prognosis, sepsis

## Abstract

**Background:**

The Geriatric Nutritional Risk Index (GNRI) is a validated nutritional assessment tool predictive of outcomes in elderly patients, yet its prognostic value in adult cancer patients with sepsis remains unexplored.

**Methods:**

This retrospective cohort study included adult cancer patients with sepsis from two sources: 523 patients from Guangxi Medical University Cancer Hospital ICU (2013–2024) and 4,447 patients from the MIMIC-IV database (2008–2019). Cox multivariable regression was used to analyze the association between GNRI and 28-day/60-day all-cause mortality during ICU stay. Restricted cubic splines (RCS), proportional hazards (PH) assumption testing, and landmark analyses were performed to assess linearity and temporal stability. Predictive performance was evaluated using area under the receiver operating characteristic (AUROC) curve, calibration curves with bootstrap resampling, decision curve analysis (DCA), integrated discrimination improvement (IDI), and net reclassification improvement (NRI). Internal cross-validation was conducted to validate model stability and correct for overfitting.

**Results:**

In Cox multivariable regression analysis, GNRI levels were independently associated with reduced mortality in both cohorts. Restricted cubic spline (RCS) curves demonstrated a linear dose–response relationship between GNRI and mortality. PH testing and landmark analysis confirmed the time-independent prognostic value of GNRI. Subgroup analyses confirmed the robustness of these findings. The model achieved excellent discriminative ability (AUC 0.906 in the Guangxi cohort and 0.810 in the MIMIC-IV cohort for 28-day mortality). Calibration curves showed excellent agreement between predicted and observed probabilities. DCA confirmed favorable net clinical benefit across a wide range of risk thresholds. IDI and NRI verified that GNRI significantly improved risk classification. Internal cross-validation showed stable optimism-corrected C-indices with minimal overfitting.

**Conclusion:**

Among adult patients with cancer and sepsis, a lower GNRI is associated with increased short- and medium-term all-cause mortality. GNRI showed excellent discriminative ability, good calibration, favorable clinical net benefit, significant improvement in risk classification, and robust stability in internal validation, with consistent performance in external validation. GNRI may serve as a simple, reliable prognostic tool for risk stratification and early nutritional intervention in this high-risk population.

## Introduction

The current international Sepsis-3 consensus defines sepsis as life-threatening organ dysfunction resulting from a dysregulated host response to an infection (1). It is estimated to affect about 30 million people worldwide each year and is associated with a high mortality of approximately 6 million (2). In sepsis, a dysregulated immune response to infection triggers a hyperinflammatory state, characterized by a massive release of pro-inflammatory factors that induce systemic damage (3). This, combined with the synergistic effect of systemic inflammatory response and dysregulated oxidative stress, ultimately progresses to multiple organ dysfunction and failure (4). Current clinical observations indicate that among sepsis patients admitted to the ICU, both low albumin levels (5) and a low body mass index (BMI) at admission (6) are associated with increased mortality. Conversely, a higher BMI is correlated with improved survival rates (7). These findings underscore the profound impact of nutritional status on sepsis outcomes. Patients with malignancy may progressively develop cancer cachexia (8) or conditions like sarcopenia (9), leading to significant weight loss. Substantial evidence indicates that malnutrition in cancer patients is associated with increased postoperative complications, prolonged hospitalization, and higher mortality (10), underscoring its significant negative impact on clinical outcomes. The modern paradigm of specialized oncology care centralizes treatment for cancer patients. A significant clinical challenge in this setting is that anticancer therapies can cause immunosuppression, predisposing patients to serious infections which may progress to sepsis and require intensive care. This reality underscores the imperative to focus on the large and vulnerable cohort of patients with cancer and sepsis.

Previous studies have investigated the prognostic value of nutritional indices in sepsis, including the Geriatric Nutritional Risk Index (GNRI) (11). The GNRI is a modified version of the Nutritional Risk Index (NRI) that incorporates body weight, height, and serum albumin levels. It is specifically designed to assess nutritional status in elderly individuals with pathological conditions (12). Recent studies have established significant associations between the GNRI and various diseases in the elderly, including cardiovascular disease (13), cerebrovascular disease (14), cancer (10), chronic lung disease (15), chronic heart failure (16), thrombocytopenia (17), diabetes (18), chronic kidney failure (19), and liver cirrhosis (20). The GNRI offers distinct advantages over individual nutritional parameters, such as serum albumin or body mass index (BMI), a point that will be elaborated upon in the discussion. Furthermore, its application and validation have been primarily investigated in geriatric populations.

However, the association between the GNRI and prognosis remains unexplored in the specific population of patients with both malignancy and sepsis. We hypothesized that GNRI could independently predict short-term mortality in this specific patient population.

The novelty of the present study lies in its unique study population, comprehensive validation strategy, and clinical generalizability compared with prior research. First, previous sepsis-GNRI studies focused only on elderly patients, single-center cohorts, or general ICU populations, and none specifically enrolled patients with cancer and sepsis. Second, existing cancer-GNRI investigations primarily examined non-septic cancer patients or those outside the ICU setting, lacking data on critically ill cancer patients with sepsis. Third, prior nutritional risk studies using large databases such as MIMIC included general ICU populations but did not simultaneously focus on the dual high-risk group of patients with both cancer and sepsis. By combining a specialized cancer ICU cohort with external validation in a large international database, this study is the first to systematically evaluate the prognostic value of GNRI in adult cancer patients with sepsis across different age groups and clinical settings.

## Methods

### Ethics

The first part of this study was approved by the Ethics Committee of the Guangxi Medical University Cancer Hospital, China, in the fourth quarter of 2025 (Ethics Approval No.: KY20251072). All protected patient health information was anonymized and posed no risk to patients. Accordingly, the local ethics committee granted a waiver of informed consent for this retrospective study. The second part of the data was based on the MIMIC-IV database (version 3.1), developed and maintained by the Massachusetts Institute of Technology (MIT) Computational Physiology Laboratory. We completed the required coursework for database access and obtained certification for He Yili (Certification No.: 56396864). This manuscript was prepared in accordance with the Strengthening the Reporting of Observational Studies in Epidemiology (STROBE) guidelines.

### Study design and population

This study first utilized inpatient data from a tertiary provincial-level medical center in Southwest China, including patients aged ≥18 years with underlying malignancy and sepsis admitted to the ICU between 2013 and 2024 from provinces such as Guangxi, Guizhou, and Yunnan. The second analysis examined data from Beth Israel Deaconess Medical Center in Boston, Massachusetts, located in the New England region of the United States. An identical patient population was enrolled from ICU admissions between 2008 and 2019. Eligible participants met the following criteria: (1) admission to the ICU; (2) age ≥18 years; (3) diagnosis of both malignancy and sepsis. Patients were excluded if they met any of the following: (1) repeated ICU admissions (only the first admission was included); (2) ICU length of stay <24 h; (3) missing outcome data; or (4) missing measurements for height, weight, or albumin levels.

### Data collection and outcomes

The primary outcome was all-cause mortality in the ICU, assessed at 28-day and 60-day timepoints following ICU admission. Independent variables tested as predictors included: baseline demographic data (sex, age, height, weight, etc.), scoring systems (APACHE-II, SOFA, and activities of daily living scores), vital signs, comorbidities (hypertension, diabetes, myocardial infarction, heart failure, stroke, chronic lung disease, severe liver disease, peptic ulcer disease, metastatic solid tumor, etc.), treatment measures (surgical procedures, anti-tumor therapy, mechanical ventilation, vasoactive drug use, blood purification, maintenance corticosteroid therapy, etc.), and laboratory results (complete blood count, blood gas analysis, liver function, renal function, coagulation profile, etc.), as well as hospital and ICU length of stay.

Covariate selection was performed using the following criteria: (1) clinical relevance; (2) consistency with covariates reported in previous GNRI-related studies; (3) inclusion of variables with *p* < 0.1 in univariate analysis; (4) inclusion of variables that altered the effect size of GNRI on mortality by > 10% after adjustment; and (5) limitation of the total number of covariates based on sample size and outcome event count.

The GNRI was calculated using the following formula:

GNRI = 1.489 × albumin (g/L) + [41.7 × (actual body weight/ideal body weight)].

The ideal body weight (WLo) was estimated based on height and gender using the Lorentz equations as follows:

- For males: WLo (kg) = height (cm) - 100 - [(height - 150)/4]
- For females: WLo (kg) = height (cm) - 100 - [(height - 150)/2.5]

### Missing data

No missing data were observed for the primary study measures, including height, weight, serum albumin level, and 28-day and 60-day mortality counts. The rates of missing data for other variables were as follows: (The Guangxi Cancer Hospital Intensive Care Database) APACHE-II score 6.12%, SOFA score 10.52%, BNP 21.03%, PCT 13.58%, CRP 10.33%, IL-6 46.46%, P/F ratio 10.13%, PCO₂ 5.54%, pH 5.54%, PO₂ 5.54%, total CO₂ 18.36%, base excess 7.07%, lactate 11.28%, glucose 14.72%. (MIMIC-IV database) lymphocytes/monocytes/neutrophils 35.17%, ALT 38.61%, AST 38.43%, total bilirubin 38.41%, creatine kinase (CK) 69.60%, CK-MB 69.71%, lactate 28.54%, P/F ratio 33.3%, prothrombin time (PT) 5.6%, activated partial thromboplastin time (APTT) 6.03%, PO₂/PCO₂ 20.8%, and D-dimer 98.52%. All other variables had missing data rates of less than 5% or were complete. Variables with a missing data proportion exceeding 45% were excluded from modeling. For the remaining variables with missingness ≤ 45%, multiple imputation was performed to handle missing values.

### Statistical analysis

The normality of continuous variables was assessed using the Kolmogorov–Smirnov test. Normally distributed variables are presented as mean ± standard deviation and compared using the independent samples t-test. Non-normally distributed variables are expressed as median with interquartile range (IQR) and compared using the Mann–Whitney *U* test (for two groups) or Kruskal–Wallis test (for multiple groups). Categorical variables are summarized as frequencies and percentages, with comparisons performed using the chi-square test or Fisher’s exact test as appropriate.

The independent association between GNRI and 28-day/60-day mortality was assessed using multivariable Cox regression analysis, with multiple models constructed to evaluate the robustness of this association. Forest plots were utilized to display interaction effects between GNRI and specific patient subgroups. The potential nonlinear relationship between GNRI and all-cause mortality was examined using RCS. The proportional hazards assumption was verified for both the GNRI variable and the full model, with residual plots providing visual assessment of this assumption. The predictive performance of GNRI for mortality in patients with malignancy and sepsis was evaluated by calculating the AUROC, along with sensitivity and specificity. The Youden index was employed to determine the optimal cutoff value and assess overall predictive accuracy.

All analyses were performed using R statistical software (version 4.3.2; R Foundation for Statistical Computing) and Free Statistics software (version 2.1). A two-tailed *p*-value < 0.05 was considered statistically significant for all analyses.

## Results

### Study population

**Figure 1:**
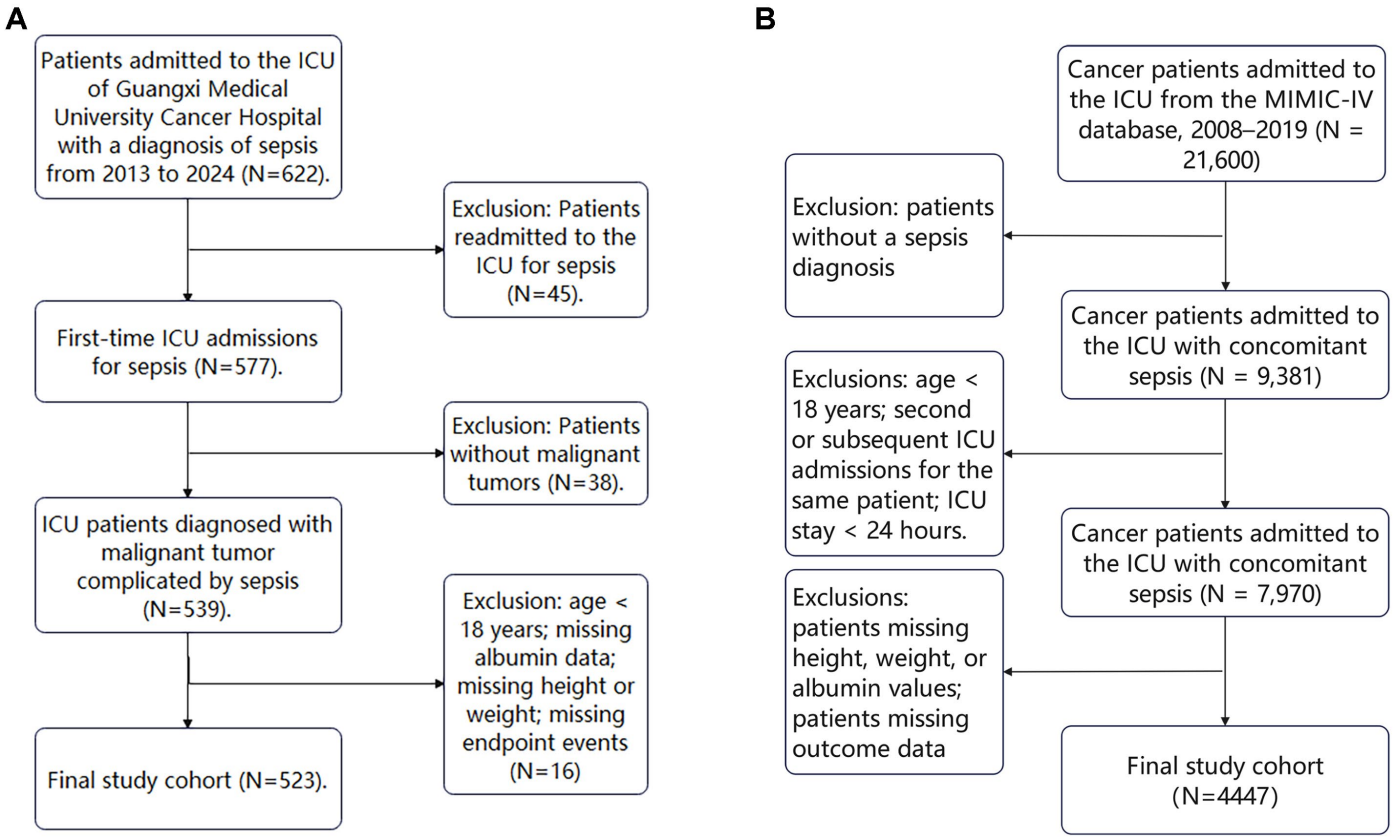
**(A)** Flow diagram of subject selection (Guangxi Cancer Hospital). **(B)** Flow diagram of subject selection (MIMIC-IV).

This study ultimately included 523 adult patients diagnosed with cancer and sepsis who were admitted to the ICU from Guangxi Medical University Cancer Hospital (Figure 1A). The cohort consisted of 323 males (61.8%) and 200 females (38.2%), with a mean age of 59.3 ± 12.6 years. The mean height was 162.4 ± 7.8 cm, and the actual body weight was 57.5 ± 10.6 kg. The resulting mean BMI fell within the normal range, indicating that the study population was not obese. This specialized patient population exhibited high mortality rates: 37.1% at 28 days and 48.4% at 60 days. Patients with higher GNRI values showed elevated levels of mean arterial pressure, incidence of coronary heart disease, stroke events, hemoglobin, platelets, globulin, creatine kinase, and creatinine. Conversely, these patients demonstrated lower rates of metastatic solid tumors and reduced levels of PCT, lactate, prothrombin time, and activated partial thromboplastin time (Supplementary Table 1).

A total of 4,447 patients from the US MIMIC-IV 3.1 database were included (Figure 1B), comprising 2,670 males (60%) and 1,777 females (40%), with a mean age of 71.0 ± 12.3 years. The mean height was 169.2 ± 10.5 cm, and mean actual body weight was 81.0 ± 21.7 kg. The mean BMI indicated an obese population. Mortality rates in this specific patient cohort were 25.7% at 28 days and 31.2% at 60 days. Patients with higher GNRI levels exhibited lower heart rate, fewer cases of stroke, fewer cases of metastatic solid tumors, lower rates of corticosteroid therapy, reduced platelet levels, decreased anion gap, and lower PT levels (Supplementary Table 1).

### Association between GNRI and mortality

To examine the relationship between GNRI and mortality, five progressively adjusted models were constructed by sequentially adding covariates (Table 1). In the Guangxi Cancer Hospital (China) database, GNRI was analyzed both as a continuous variable and as a categorical variable divided into tertiles. As a continuous variable, GNRI showed a consistent inverse association with 28-day mortality across all models: unadjusted (HR = 0.98, 95% CI: 0.97–0.99, *p* = 0.003), Model 1 (HR = 0.98, 95% CI: 0.96–1.00, *p* = 0.024), Model 2 (HR = 0.98, 95% CI: 0.96–1.00, *p* = 0.037), Model 3 (HR = 0.97, 95% CI: 0.95–0.99, p = 0.003), Model 4 (HR = 0.97, 95% CI: 0.95–0.99, *p* = 0.011), and Model 5 (HR = 0.97, 95% CI: 0.95–0.99, *p* = 0.005), with each unit increase in GNRI associated with a 3% reduction in mortality risk in the fully adjusted model. When analyzed by tertiles, the highest GNRI tertile consistently exhibited a lower mortality risk compared with the lowest tertile across all models: unadjusted (HR = 0.61, 95% CI: 0.43–0.86, *p* = 0.004), Model 1 (HR = 0.60, 95% CI: 0.40–0.92, *p* = 0.020), Model 2 (HR = 0.59, 95% CI: 0.38–0.93, *p* = 0.022), Model 3 (HR = 0.50, 95% CI: 0.31–0.79, *p* = 0.003), Model 4 (HR = 0.55, 95% CI: 0.34–0.88, *p* = 0.013), and Model 5 (HR = 0.51, 95% CI: 0.31–0.86, *p* = 0.012). A consistent trend was observed for 60-day mortality, with detailed results presented in Table 1.

**Table 1:** Risk of 28-day and 60-day mortality according to GNRI (Guangxi Cancer Hospital database).

Variable	Non-adjusted		Model 1		Model 2	
	Crude HR (95%CI)	Crude *p*-value	Adj. HR (95%CI)	Adj. *p*-value	Adj. HR (95%CI)	Adj. *p*-value
28-day mortality						
GNRI	0.98 (0.97 ~ 0.99)	0.003	0.98 (0.96 ~ 1.00)	0.024	0.98 (0.96 ~ 1.00)	0.037
Tertile of GNRI						
T1	Reference		Reference		Reference	
T2	0.68 (0.48 ~ 0.95)	0.024	0.66 (0.46 ~ 0.94)	0.023	0.73 (0.5 ~ 1.05)	0.086
T3	0.61 (0.43 ~ 0.86)	0.004	0.6 (0.4 ~ 0.92)	0.02	0.59 (0.38 ~ 0.93)	0.022
60-day mortality						
GNRI	0.98 (0.97 ~ 0.99)	0.002	0.98 (0.97 ~ 1.00)	0.048	0.98 (0.97 ~ 1.00)	0.051
Tertile of GNRI						
T1	Reference		Reference		Reference	
T2	0.7 (0.52 ~ 0.95)	0.02	0.71 (0.52 ~ 0.97)	0.029	0.77 (0.56 ~ 1.06)	0.11
T3	0.66 (0.49 ~ 0.89)	0.007	0.71 (0.49 ~ 1.03)	0.071	0.7 (0.48 ~ 1.03)	0.071

Variable	Model 3		Model 4		Model 5	
	Adj. HR (95%CI)	Adj. *p*-value	Adj. HR (95%CI)	Adj. *p*-value	Adj. HR (95%CI)	Adj. *p*-value
28-day mortality						
GNRI	0.97 (0.95 ~ 0.99)	0.003	0.97 (0.95 ~ 0.99)	0.011	0.97 (0.95 ~ 0.99)	0.005
Tertile of GNRI						
T1	Reference		Reference		Reference	
T2	0.7 (0.48 ~ 1.02)	0.061	0.68 (0.46 ~ 1.00)	0.051	0.64 (0.41 ~ 0.98)	0.038
T3	0.5 (0.31 ~ 0.79)	0.003	0.55 (0.34 ~ 0.88)	0.013	0.51 (0.31 ~ 0.86)	0.012
60-day mortality						
GNRI	0.98 (0.96 ~ 0.99)	0.005	0.98 (0.96 ~ 1.00)	0.016	0.98 (0.96 ~ 0.99)	0.01
Tertile of GNRI						
T1	Reference		Reference		Reference	
T2	0.72 (0.51 ~ 1.00)	0.051	0.71 (0.5 ~ 1.00)	0.048	0.66 (0.45 ~ 0.97)	0.032
T3	0.58 (0.38 ~ 0.86)	0.007	0.63 (0.41 ~ 0.96)	0.031	0.61 (0.39 ~ 0.96)	0.034

Model 1 adjusted for age, gender, BMI, heart rate, MAP and Spo2.

Model 2 adjusted for model 1 plus APACHE-II score, SOFA score, DM+CC, CAD, HF, stroke, SLD, CLD.

Model 3 adjusted for model 2 plus ATT (chemo/TT/IO), RT, surgery, MV, vasopressin, RRT.

Model 4 adjusted for model 3 plus WBC, neutrophils; lymphocytes; HGB, PLT, PCT, NTpro-BNP, D-dimer and CRP.

Model 5 adjusted for model 3 plus AG, P/F, PT, ALT, AST, CK, CK-MB, Ccr and Mg++.

GNRI, Geriatric Nutritional Risk Index; BMI, body mass index; MAP, mean arterial pressure; Spo2, peripheral oxygen saturation; APACHE-II, Acute Physiology And Chronic Health Evaluation II; SOFA, sequential organ failure assessment; DM+CC, diabetes mellitus with complications; CAD, coronary artery disease; HF, heart failure; SLD, severe liver disease; CLD, chronic lung disease; ATT, antitumor therapy; Chemo, chemotherapy; TT, targeted therapy; IO, immunooncology therapy; RT, radiotherapy; MV, mechanical ventilation; RRT, renal replacement therapy; WBC, white blood cell; HGB, hemoglobin; PLT, platelet; PCT, procalcitonin; NTpro-BNP, N-terminal pro-B-type natriuretic peptide; CRP, C-reactive protein; AG, anion gap; P/F, PaO₂/FiO₂ ratio; PT, prothrombin time; ALT, alanine aminotransferase; AST, aspartate aminotransferase; CK, creatine kinase; CK-MB, creatine Kinase-Myocardial band; SCr, serum creatinine.

In the MIMIC-IV database, GNRI analyzed as a continuous variable (from univariate analysis through Model 5) consistently showed an association with mortality (HR = 0.99, 95% CI: 0.99–0.99, *p* < 0.001). When categorized into tertiles and incorporated into the models, the highest tertile compared with the lowest tertile demonstrated reduced mortality (HR = 0.70, 95% CI: 0.61–0.82, *p* < 0.001). For 60-day mortality, GNRI as a continuous variable (from univariate analysis through Model 5) similarly showed consistent results (HR = 0.99, 95% CI: 0.99–0.99, *p* < 0.001), while analysis by tertiles revealed that the highest tertile compared with the lowest tertile was associated with reduced mortality (HR = 0.68, 95% CI: 0.60–0.78, *p* < 0.001) (Table 2).

**Table 2:** Risk of 28-day and 60-day mortality according to GNRI (MIMIC-IV database).

Variable	Non-adjusted		Model 1		Model 2	
	Crude HR (95%CI)	Crude *p*-value	Adj. HR (95%CI)	Adj. *p*-value	Adj. HR (95%CI)	Adj. *p*-value
28-day mortality						
GNRI	0.99 (0.99 ~ 0.99)	<0.001	0.99 (0.99 ~ 0.99)	<0.001	0.99 (0.99 ~ 0.99)	<0.001
Tertile of GNRI						
T1	Reference		Reference		Reference	
T2	0.76 (0.66 ~ 0.87)	<0.001	0.76 (0.66 ~ 0.87)	<0.001	0.76 (0.66 ~ 0.87)	<0.001
T3	0.7 (0.61 ~ 0.82)	<0.001	0.7 (0.61 ~ 0.82)	<0.001	0.7 (0.61 ~ 0.82)	<0.001
Trend.test	0.83 (0.77 ~ 0.89)	<0.001	0.83 (0.77 ~ 0.89)	<0.001	0.83 (0.77 ~ 0.89)	<0.001
60-day mortality						
GNRI	0.99 (0.99 ~ 0.99)	<0.001	0.99 (0.99 ~ 0.99)	<0.001	0.99 (0.99 ~ 0.99)	<0.001
Tertile of GNRI						
T1	Reference		Reference		Reference	
T2	0.79 (0.7 ~ 0.89)	<0.001	0.79 (0.7 ~ 0.89)	<0.001	0.79 (0.7 ~ 0.89)	<0.001
T3	0.68 (0.6 ~ 0.78)	<0.001	0.68 (0.6 ~ 0.78)	<0.001	0.68 (0.6 ~ 0.78)	<0.001
Trend.test	0.82 (0.77 ~ 0.88)	<0.001	0.82 (0.77 ~ 0.88)	<0.001	0.82 (0.77 ~ 0.88)	<0.001

Variable	Model 3		Model 4		Model 5	
	Adj. HR (95%CI)	Adj. *p*-value	Adj. HR (95%CI)	Adj. *p*-value	Adj. HR (95%CI)	Adj. *p*-value
28-day mortality						
GNRI	0.99 (0.99 ~ 0.99)	<0.001	0.99 (0.99 ~ 0.99)	<0.001	0.99 (0.99 ~ 0.99)	<0.001
Tertile of GNRI						
T1	Reference		Reference		Reference	
T2	0.76 (0.66 ~ 0.87)	<0.001	0.76 (0.66 ~ 0.87)	<0.001	0.76 (0.66 ~ 0.87)	<0.001
T3	0.7 (0.61 ~ 0.82)	<0.001	0.7 (0.61 ~ 0.82)	<0.001	0.7 (0.61 ~ 0.82)	<0.001
Trend.test	0.83 (0.77 ~ 0.89)	<0.001	0.83 (0.77 ~ 0.89)	<0.001	0.83 (0.77 ~ 0.89)	<0.001
60-day mortality						
GNRI	0.99 (0.99 ~ 0.99)	<0.001	0.99 (0.99 ~ 0.99)	<0.001	0.99 (0.99 ~ 0.99)	<0.001
Tertile of GNRI						
T1	Reference		Reference		Reference	
T2	0.79 (0.7 ~ 0.89)	<0.001	0.79 (0.7 ~ 0.89)	<0.001	0.79 (0.7 ~ 0.89)	<0.001
T3	0.68 (0.6 ~ 0.78)	<0.001	0.68 (0.6 ~ 0.78)	<0.001	0.68 (0.6 ~ 0.78)	<0.001
Trend.test	0.82 (0.77 ~ 0.88)	<0.001	0.82 (0.77 ~ 0.88)	<0.001	0.82 (0.77 ~ 0.88)	<0.001

Model 1 adjusted for age, gender, heart rate, MAP, apsiii score, SOFA score.

Model 2 adjusted for model 1 plus DM + CC, CAD, HF, stroke, SLD, CLD, peptic ulcer disease.

Model 3 adjusted for model 2 plus hormone, MV, vasopressin, RRT.

Model 4 adjusted for model 3 plus WBC, neutrophils; lymphocytes; HGB, PLT, AG, BUN, Scr, lactate.

Model 5 adjusted for model 3 plus P/F ratio, PO2, PCO2, PT, APTT, ALT, AST, TBIL.

GNRI, Geriatric Nutritional Risk Index; BMI, body mass index; MAP, mean arterial pressure; apsiii score, Acute Physiology Score III; SOFA, sequential organ failure assessment; DM + CC, diabetes mellitus with complications; CAD, coronary artery disease; HF, heart failure; SLD: severe liver disease; CLD, chronic lung disease; MV, mechanical ventilation; RRT, renal replacement therapy; WBC, white blood cell; HGB, hemoglobin; PLT, platelet; AG, anion gap; BUN, blood urea nitrogen; SCr, serum creatinine; P/F, PaO₂/FiO₂ ratio; PT, prothrombin time; APTT, activated partial thromboplastin time; ALT, alanine aminotransferase; AST, aspartate aminotransferase; TBIL, total bilirubin.

These findings confirm that GNRI is inversely associated with short-term mortality in adult patients with cancer and sepsis.

### Restricted cubic splines analysis

**Figure 2:**
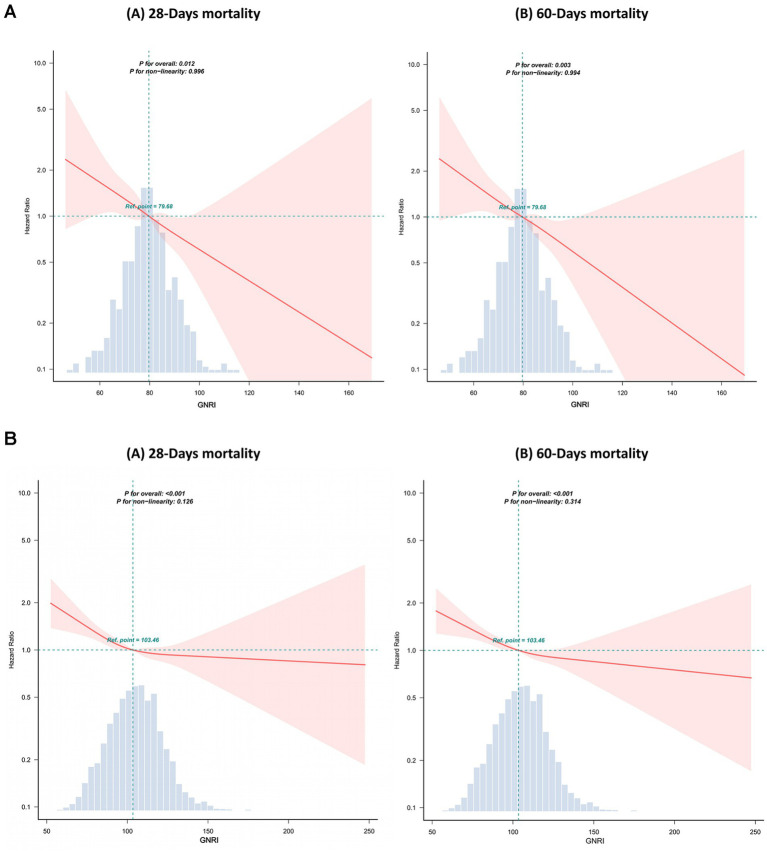
**(A)** Curve fitting of GNRI and mortality (Guangxi Cancer Hospital). Adjusted covariates included general baseline characteristics, critical illness severity scores, comorbidities, key therapeutic interventions, and laboratory findings. **(B)** Curve fitting of GNRI and mortality (MIMIC-IV). Adjusted covariates included general baseline characteristics, critical illness severity scores, comorbidities, key therapeutic interventions, and laboratory findings.

Restricted cubic splines were fitted using the same set of covariates adjusted for in the primary models, including baseline characteristics, comorbidities, critical illness severity scores, primary ICU treatments, and key laboratory parameters. In the Guangxi Cancer Hospital cohort: for the 28-day mortality model, P for overall = 0.012 and P for non-linearity = 0.996; for the 60-day mortality model, P for overall = 0.003 and P for non-linearity = 0.994 (Figure 2A). In the US MIMIC-IV cohort: for the 28-day mortality model, p for overall<0.001 and p for non-linearity = 0.126; for the 60-day mortality model, P for overall<0.001 and P for non-linearity = 0.314 (Figure 2B). These results demonstrate a consistent linear dose–response relationship between GNRI and short- to medium-term mortality in adult patients with cancer and sepsis.

### Proportional hazards assumption and landmark survival analysis

The proportional hazards (PH) assumption was evaluated using Schoenfeld residuals. In the Guangxi cohort, both GNRI alone and the global model satisfied the PH assumption for 28-day and 60-day mortality (all *p* > 0.05; Supplementary Tables 2, 3, Supplementary Figures 1A,B). In the MIMIC-IV cohort, GNRI itself met the PH assumption (all *p* > 0.05), whereas the global test violated the assumption (all *p* < 0.001; Supplementary Tables 4, 5, Supplementary Figures 1C,D).

Landmark analyses at day 7 and day 14 confirmed that the inverse association between GNRI and mortality remained stable and time-independent throughout follow-up (Supplementary Tables 6, 7).

### Subgroup analyses

**Figure 3:**
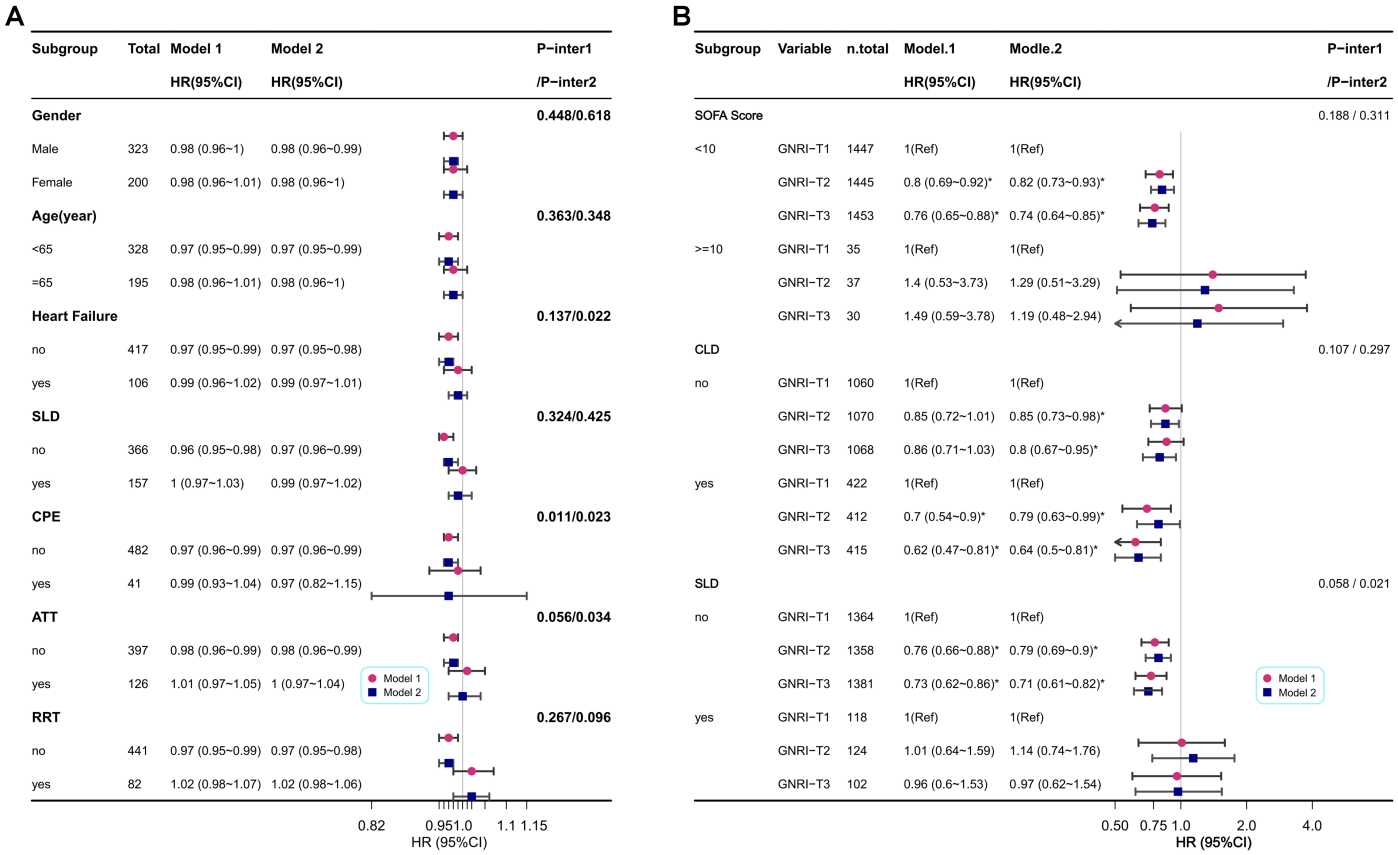
**(A)** MForest plot of GNRI and covariates (Guangxi Cancer Hospital). SLD, severe liver disease; CPE, chronic pulmonary embolism; ATT, antitumor therapy; chemo, chemotherapy; RRT, renal replacement therapy; P-inter1, P for interaction in model 1; P-inter2, P for interaction in model 2; Model 1, 28-day mortality model; Model 2, 60-day mortality model. **(B)** MForest plot of GNRI and covariates (MIMIC-IV). CLD, chronic lung disease; SLD, severe liver disease; P-inter1, P for interaction in model 1; P-inter2, P for interaction in model 2; Model 1, 28-day mortality model; Model 2, 60-day mortality model; *p < 0.05.

**Figure 4:**
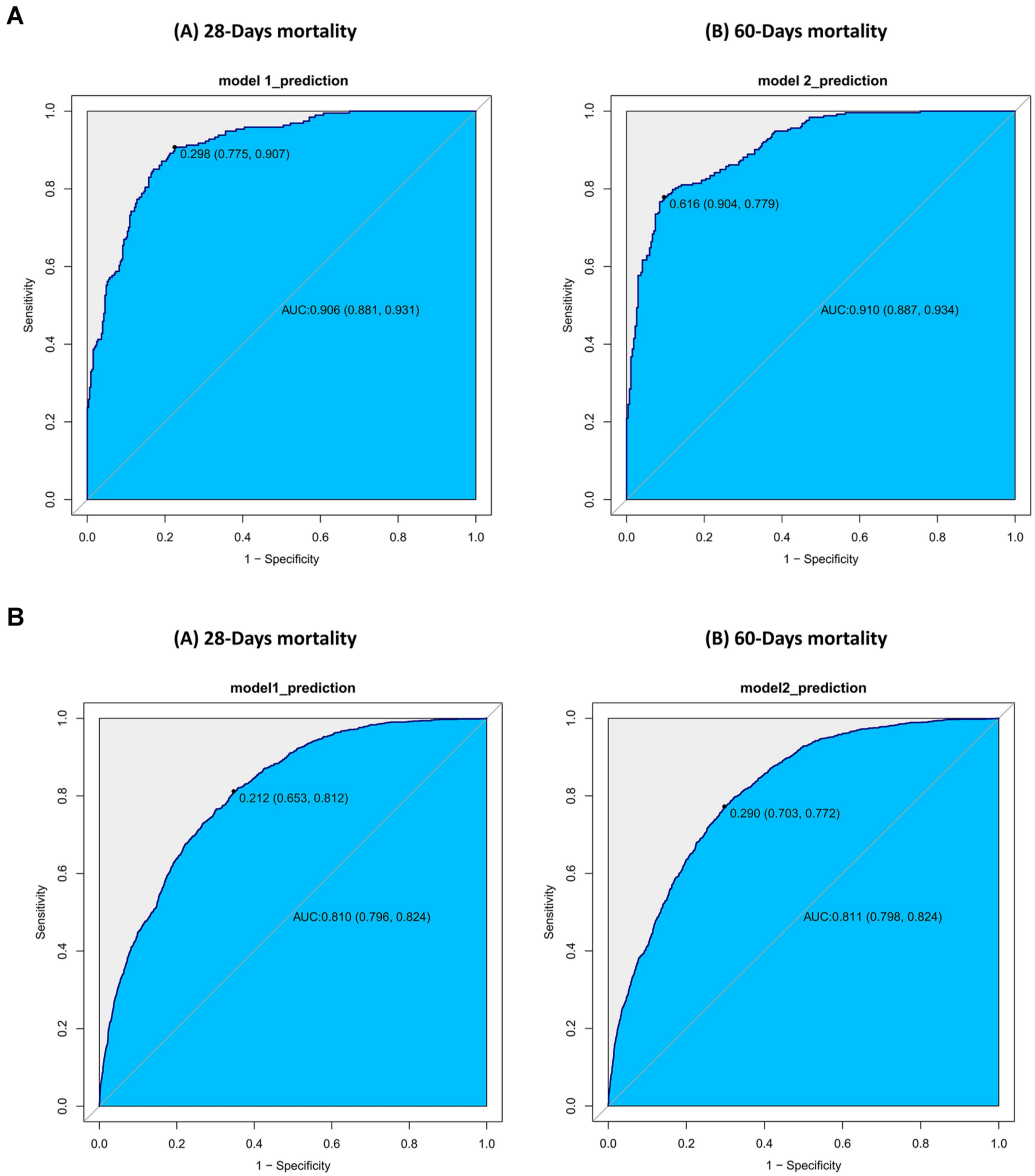
**(A)** ROC curve of GNRI and mortality (Guangxi Cancer Hospital). **(B)** ROC curve of GNRI and mortality (MIMIC-IV).

Subgroup analyses are presented in Figures 3, 4. Stratification by baseline characteristics, severity scores, clinical treatments, comorbidities, and laboratory parameters revealed significant interaction effects in several specific subgroups: in the Guangxi Cancer Hospital database, patients with comorbid heart failure (P for interaction = 0.022 in the 60-day mortality model), those with a history of pulmonary embolism (P for interaction = 0.011 in the 28-day model and 0.023 in the 60-day model), and patients receiving antitumor therapy (P for interaction = 0.034 in the 60-day mortality model). In the US MIMIC-IV database, the severe liver disease subgroup (P for interaction = 0.021 in the 60-day mortality model) represented a special population.

### Area under the ROC curve analysis

The predictive performance of GNRI for 28-day and 60-day all-cause mortality in patients with cancer and sepsis was evaluated using receiver operating characteristic (ROC) curve analysis. In the Guangxi Cancer Hospital database, the area under the ROC curve (AUC) was 0.906 for 28-day mortality and 0.910 for 60-day mortality (Figure 4A). At the optimal cutoff points, the Youden indices were 0.6823 and 0.6824, respectively (Table 3). In the US MIMIC-IV database, the AUC values were 0.810 and 0.811, respectively (Figure 4B). At the optimal cutoff values, the Youden indices for the two metrics were 0.465 and 0.4748, respectively (Table 4).

**Table 3:** Information of receiver operating characteristic curve in Figure 4A.

Variable	AUC	95%CI	Threshold	Sensitivity	Specificity	Youden
Prediction for 28 days	90.6%	88.1 ~ 93.1%	0.2975	0.9072	0.7751	0.6823
Prediction for 60 days	91.0%	88.7 ~ 93.4%	0.6164	0.7787	0.9037	0.6824

AUC, area under the curve.

**Table 4:** Information of receiver operating characteristic curve in Figure 4B.

Variable	AUC	95%CI	Threshold	Sensitivity	Specificity	Youden
Prediction for 28 days	81.0%	79.6 ~ 82.4%	0.2115	0.8119	0.6531	0.465
Prediction for 60 days	81.1%	79.8 ~ 82.4%	0.2901	0.7723	0.7025	0.4748

AUC, area under the curve.

### Predictive performance, internal validation, and improvement analyses

The predictive performance of the multivariable Cox model for 28-day and 60-day all-cause mortality was further evaluated in the Guangxi cohort.

**Figure 5:**
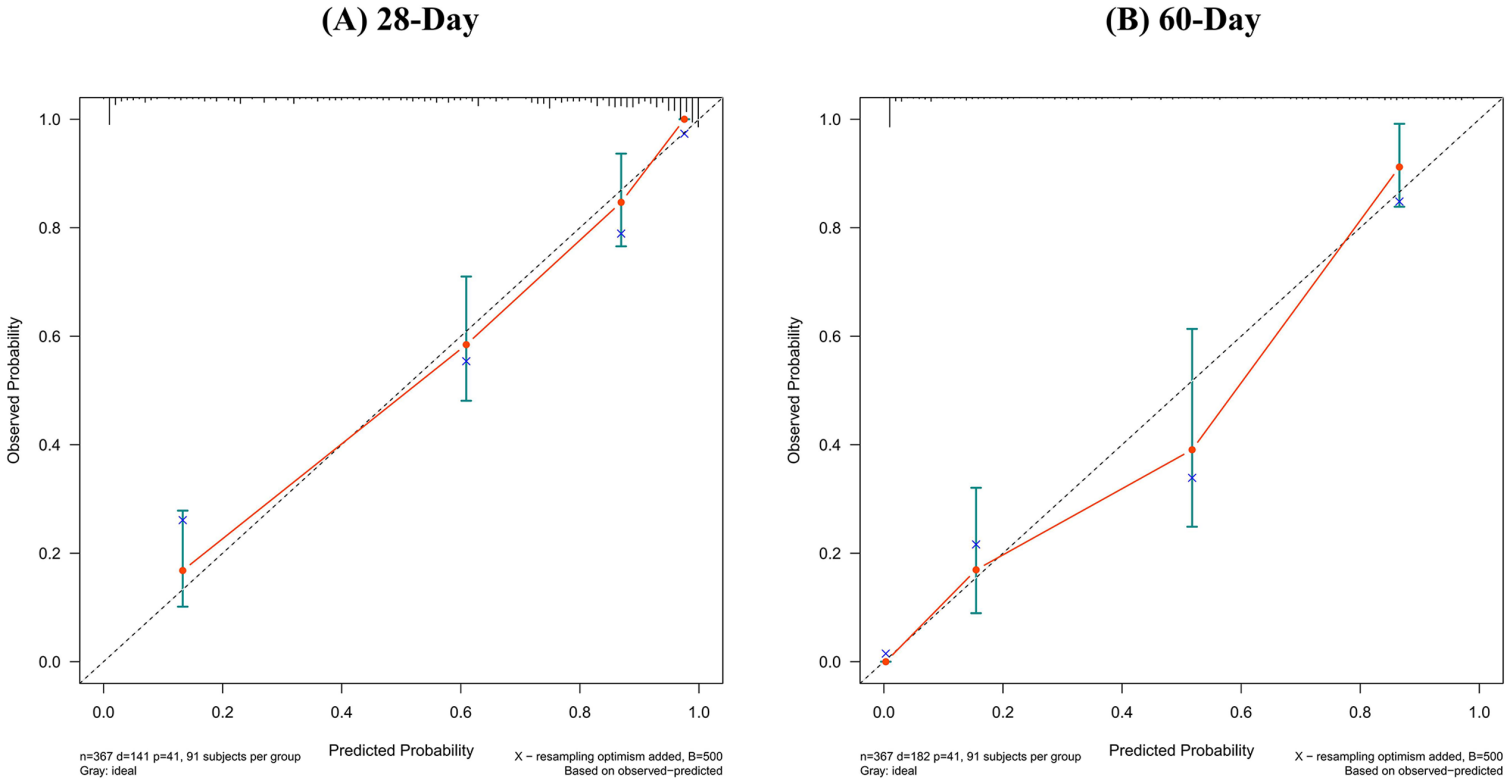
Calibration curve for predicted all-cause mortality (Guangxi Cancer Hospital). **(A)** 28 day allcause mortality calibration plot; **(B)** 60 day allcause mortality calibration plot. Dashed gray line = ideal calibration; red solid line = observed risk; blue crosses = bootstrap biascorrected predicted risk (*B* = 500); vertical bars = 95% confidence intervals. The dataset was split into four equal subgroups for calibration evaluation.

*Calibration curves* with bootstrap resampling (*B* = 500) showed excellent agreement between predicted and observed probabilities at both time points, with no significant overestimation or underestimation of mortality risk (Figure 5).

**Figure 6:**
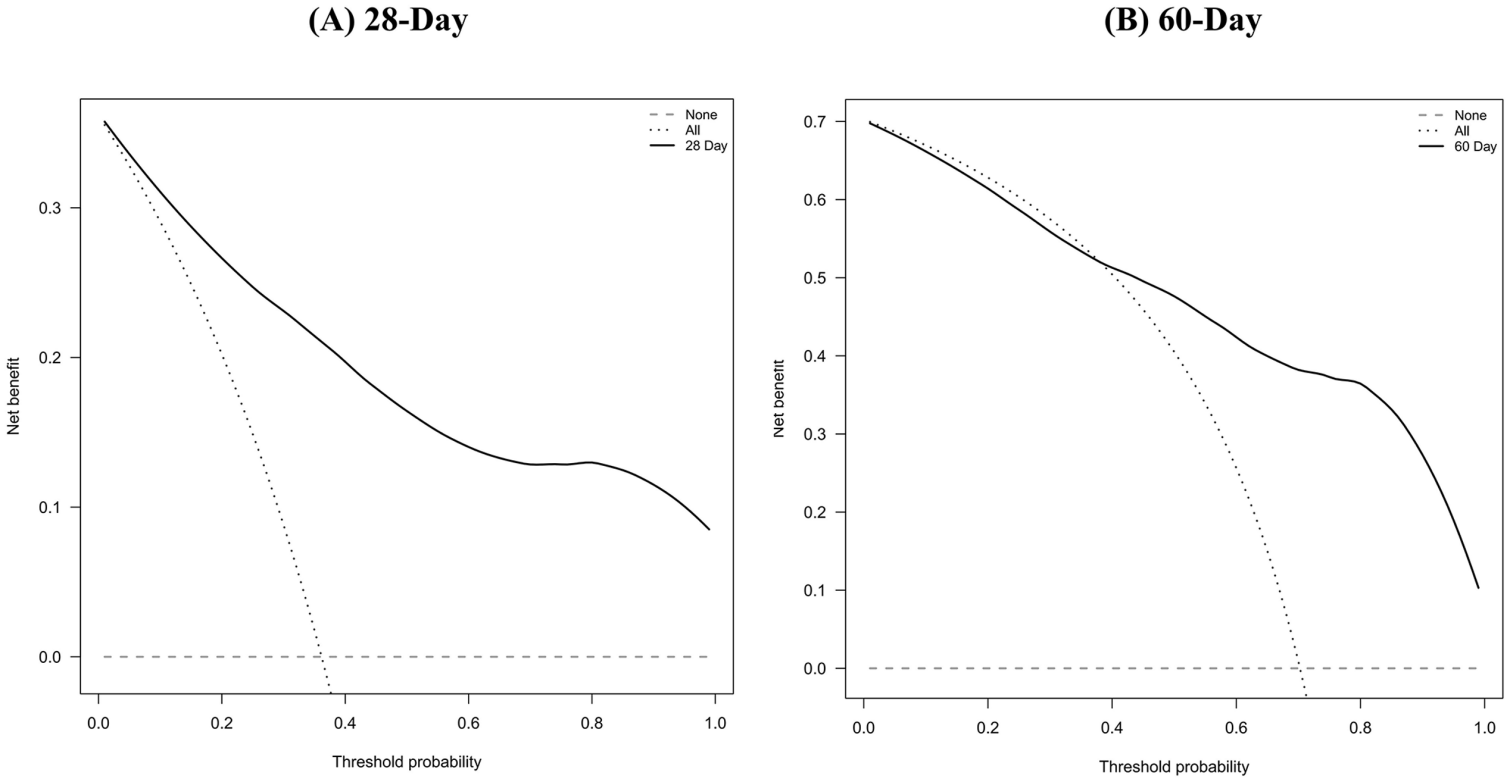
Decision curve analysis for all-cause mortality (Guangxi Cancer Hospital). **(A)** DCA of the 28 day mortality model; **(B)** DCA of the 60 day mortality model. Gray dashed line: net benefit of treating no patients; gray dotted line: net benefit of treating all patients; solid black line: net clinical benefit provided by the GNRIbased risk model at different risk threshold probabilities.

*Decision curve analysis (DCA)* demonstrated that the model provided a higher net benefit across nearly the entire range of threshold probabilities compared with the “treat all” and “treat none” strategies, confirming favorable clinical utility (Figure 6).

**Figure 7:**
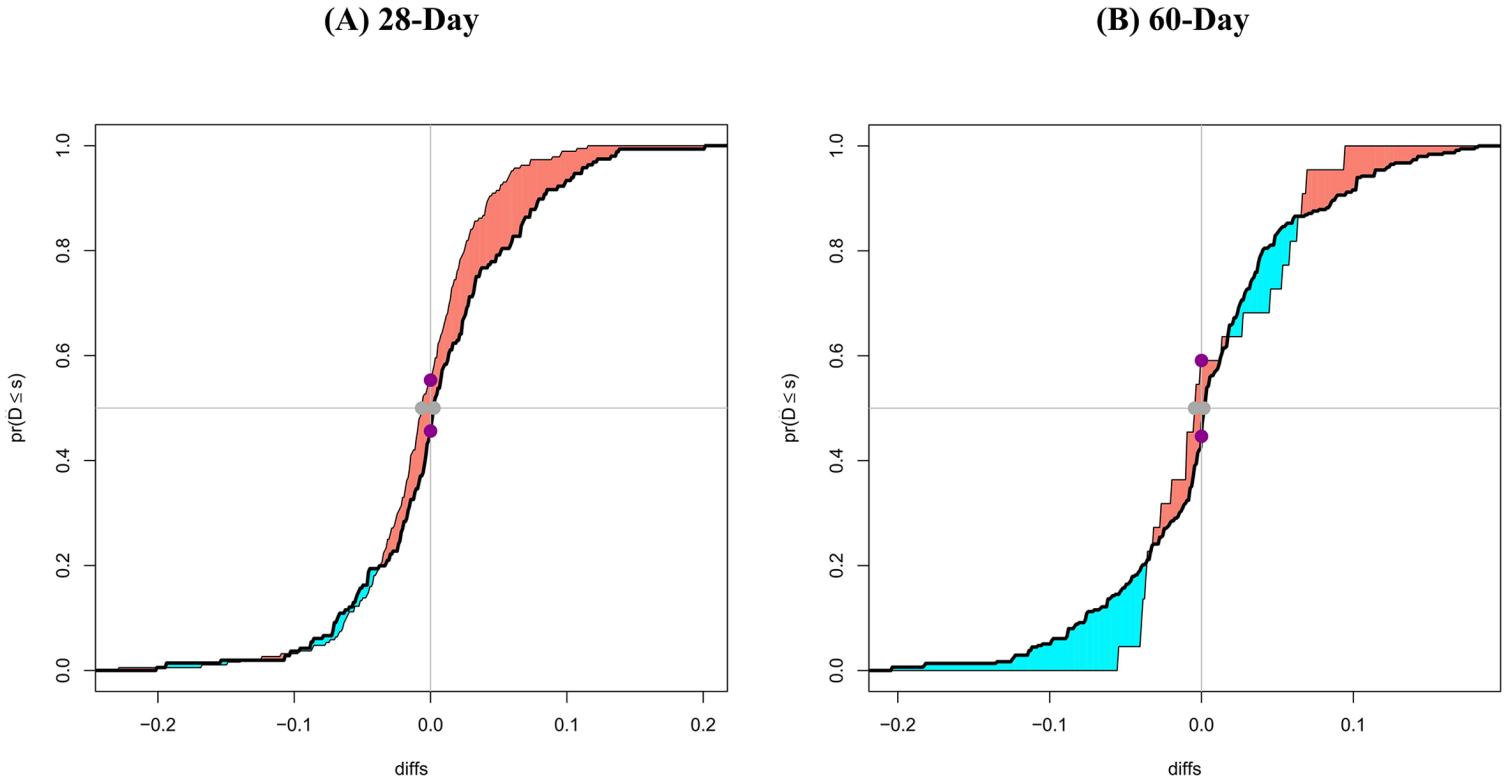
IDI analysis plot for all-cause mortality (Guangxi Cancer Hospital). **(A)** IDI plot for the 28 day mortality model; **(B)** IDI plot for the 60 day mortality model. X-axis: differences in predicted risk between the GNRIcontaining full model and the baseline model without GNRI; Y-axis: cumulative probability of risk differences. Cyan shading = reduced predicted risk after adding GNRI; red shading = increased predicted risk after adding GNRI; black line = cumulative distribution curve of probability differences.

*Integrated discrimination improvement (IDI)* analysis confirmed that the addition of GNRI significantly enhanced discriminatory capacity, with predicted probabilities consistently higher in non-survivors than in survivors (Figure 7).

*Internal cross-validation* was performed to assess model stability and correct for overfitting. For 28-day mortality, the original AUC was 0.884, and the cross-validated AUC was 0.8242. For 60-day mortality, the original AUC was 0.848, and the cross-validated AUC was 0.7716. Furthermore, internal k-fold cross-validation yielded high optimism-corrected C-indices with minimal reduction across validation folds (Supplementary Table 8), confirming robust model stability and absence of significant overfitting.

These results demonstrate that the predictive model based on GNRI exhibits high discriminative power, good calibration, favorable clinical net benefit, significant improvement in reclassification, and robust stability.

## Discussion

In this real-world study, we found that GNRI levels were inversely associated with all-cause mortality in adult cancer patients with sepsis. This association remained robust and consistent after adjusting for multiple confounding factors and across different time models. The results remained reliable even when considering interaction effects in stratified analyses of special populations. Sensitivity analysis showed that when GNRI was divided into tertiles, the highest tertile exhibited a more significant hazard ratio (HR). Furthermore, when key covariates were included in the proportional hazards assumption testing, the hazard ratio for GNRI remained constant within the 28-day and 60-day mortality windows, indicating that it did not need to be treated as a time-dependent variable or modeled with piecewise analysis. Finally, we evaluated the predictive performance of GNRI, which demonstrated excellent discriminative ability as assessed by the AUROC and Youden index.

Furthermore, comprehensive internal validation confirmed the robustness of the predictive model. Calibration curves showed excellent agreement between predicted and observed 28-day and 60-day mortality, with no substantial overestimation or underestimation. Decision curve analysis (DCA) demonstrated favorable net clinical benefit across a wide range of risk thresholds, supporting the clinical utility of GNRI-based risk stratification. Integrated discrimination improvement (IDI) and net reclassification improvement (NRI) verified that GNRI significantly enhanced risk classification beyond conventional predictors. Internal cross-validation yielded stable optimism-corrected C-indices with minimal reduction, indicating no meaningful overfitting and strong model reliability.

In previous studies, the GNRI has demonstrated independent associations with chronic wasting conditions resulting from non-malignant diseases, such as heart failure, stroke, chronic kidney disease, cirrhosis, and chronic inflammatory lung diseases. For instance, patients with liver abscesses often exhibit a pro-inflammatory state that impairs hepatic anabolic function, leading to a deterioration in nutritional status characterized by a pathological reduction in albumin synthesis (21). GNRI may reflect a chronic inflammatory state. In a study on chronic kidney disease, patients with a low GNRI score exhibited higher levels of systemic inflammatory markers, such as tumor necrosis factor-*α*, interleukin-6 (IL-6), and C-reactive protein (CRP), compared to those with higher scores (22). Plasma IL-6 levels were independently and inversely correlated with the GNRI score. Another study in older adults showed that a higher nutritional risk indicated by GNRI was associated with elevated CRP levels and reduced lymphocyte counts (23). Additionally, research involving patients with cirrhosis identified serum CRP and IL-6 levels as independent predictors of liver transplantation and mortality (24). In a 10-year cohort study of acute coronary syndrome, the malnutrition-inflammation-atherosclerosis syndrome, as a significant component, demonstrated an association with GNRI in the elderly population (25, 26). Furthermore, in patients with chronic inflammatory airway disease (CIAD), changes in inflammatory cytokines, hormone levels, and adipokines also play a role in nutritional regulation (27, 28). Malnutrition-induced inflammation and oxidative stress are central to the pathogenesis of CIAD progression, impairing immune defenses and significantly increasing the risk of pulmonary infections, which constitute a major factor leading to elevated mortality. Other pathophysiological mechanisms include persistent albumin loss in the elderly with chronic kidney disease (CKD), which is considered a cause of malnutrition and worsening renal function in CKD patients (29). Additionally, amino acids, another essential nutrient, are continuously lost (10–15 g/day) in patients with severe acute kidney injury undergoing hemodialysis, leading to a deficiency in substrates for nutritional synthesis. This contributes to low GNRI levels being associated with higher short-term mortality in these patients (30).

In older adults with cancer, a lower GNRI not only reflects a higher risk of malnutrition but also indicates immunosuppression, poor treatment adherence, and cachexia (31), highlighting the combined pathogenic effects of aging and malignancy. Consequently, studies focused on specific cancers such as gastrointestinal malignancies, lung cancer, and head and neck cancers have consistently established a strong association between GNRI and mortality (10).

A distinctive feature of the study population is the presence of sepsis, which reflects an amplified and dysregulated inflammatory response, potentially making the underlying mechanisms more pronounced than those observed in benign conditions as previously described. Second, the cohort may reflect characteristics of locally prevalent cancers (such as hepatocellular carcinoma and nasopharyngeal carcinoma). Third, this study utilized dual validation using populations from southern China in East Asia and the New England region of the United States. Fourth, the cohort has a mean age lower than that of typical geriatric populations. While the overall association trends align with prior research, these characteristics also confer relative distinctiveness to the findings.

Notably, the two cohorts exhibited substantial baseline differences, including age, body mass index (BMI), mortality rates, geographic region, ethnicity, and healthcare systems. Despite these marked disparities, the core findings remained highly consistent: lower GNRI was independently associated with higher mortality, with a clear linear relationship observed in both cohorts. Far from weakening the study’s validity, this consistency across distinct populations strengthens the robustness and generalizability of our conclusions. The fact that identical prognostic patterns emerged in two clinically and demographically diverse cohorts underscores that the association between GNRI and mortality is reliable, broadly applicable, and not confounded by population-specific characteristics.

In this study, the restricted cubic spline analysis—while fully adjusting for all covariates in the model—revealed a linear dose–response relationship between GNRI and the outcome. This finding differs from some previous studies. For example, in research utilizing the MIMIC database focusing on ICU-admitted older adults, the association between GNRI and all-cause mortality demonstrated an L-shaped nonlinear relationship, with a significant inverse correlation on the left side of the inflection point (GNRI < 78.7) and no significant association on the right side (32). A similar L-shaped pattern has also been reported in studies of elderly patients with sepsis (11). In contrast, a study utilizing the NHANES database involving cancer patients aged 40 and above continued to demonstrate a significant nonlinear L-shaped relationship between GNRI and prognosis (10). That study identified a high-risk GNRI threshold around 134, reflecting the higher nutritional demand range associated with cancer. The present study reflects the more critical nutritional status of this specific population—cancer patients with sepsis. In the Guangxi Cancer Hospital cohort, the GNRI threshold of >78.7 (or >98) remained indicative of dominant nutritional risk. With a GNRI upper limit of approximately 169 and an age range extending to adults (≥18 years), a consistently linear inverse association was observed throughout. In the New England population of the United States, the upper limit of GNRI was approximately 247, which still demonstrated a linear negative correlation, highlighting the severity of illness in cancer patients with sepsis and underscoring that nutritional risk, like other critical factors, should be continuously prioritized throughout ICU management. Notably, the population from southern China in East Asia included relatively few patients with absolute obesity (based on clinical presentation and average height/weight). This contrasts with previous studies in chronic kidney disease, where edema and overweight could coexist with malnutrition (19)—an “obesity paradox”—and also differs from cohorts with higher obesity prevalence, such as those from Western populations. This highlights the uniqueness of the study.

Low serum albumin or weight loss is associated with increased mortality in older adults, whereas extracellular fluid expansion shows an inverse relationship with these markers. Although body weight is influenced by hydration status, changes in hydration contrast sharply with variations in albumin concentration. The simultaneous use of both indicators in the GNRI helps minimize confounding effects from factors such as hydration status (33). Low albumin levels reflect protein-energy malnutrition, systemic inflammation, and compromised immune function. Conversely, a low BMI signals weight loss and diminished muscle mass, a particularly salient indicator in cancer patients due to its strong association with cachexia. Together, these components form the foundational basis of the GNRI for assessing nutritional risk (34). Numerous prior studies have established that GNRI is a superior nutritional indicator compared to albumin or BMI alone, demonstrating stronger predictive power for prognosis (33).

In the proportional hazards (PH) assumption test, results from the Chinese Guangxi cohort were satisfactory. In the MIMIC-IV database, the global test violated the PH assumption (*p* < 0.001), but GNRI as a single variable satisfied the PH assumption (*p* > 0.05). This violation is attributed to time-varying effects of other covariates, not to GNRI itself. We subsequently performed landmark analyses for validation. Using day 7 as the cutoff, the stability of GNRI was confirmed. Using day 14 as the cutoff, the effect estimates were consistent before and after the landmark. Although the *p*-value and trend test p-value were not significant after day 14, this is clinically logical: patients became more stable after day 14, mortality risk decreased, and the impact of nutritional risk weakened. The reduced statistical power was due to a smaller number of events, and this does not represent a negative finding.

To validate the robustness of the association between GNRI and all-cause mortality in cancer patients with sepsis, this study further excluded specific subpopulations to assess the consistency of the findings. Both the 28-day and 60-day mortality models demonstrated consistent trends; here we illustrate using the 60-day model. The forest plot indicates that the prognostic value of GNRI was significantly stronger in the following subgroups: patients with comorbid heart failure (P for interaction = 0.022), patients with comorbid chronic pulmonary embolism (P for interaction = 0.023), and those receiving antitumor therapy (including radiotherapy, chemotherapy, immunotherapy, or targeted therapy) (P for interaction = 0.034). Potential mechanisms are considered as follows: 1. The higher hazard ratio in the heart failure subgroup indicates that malnutrition, as reflected by a low GNRI, confers a greater mortality risk in patients with pre-existing heart failure. Heart failure and malnutrition participate in a mutually reinforcing vicious cycle. Heart failure is a catabolic state that can lead to cardiac cachexia (35), characterized by chronic inflammation, muscle wasting, hypermetabolism, and insulin resistance (36). When this condition coexists with the nutritional impairment indicated by a low GNRI, a synergistic “1 + 1 > 2” adverse effect likely occurs, markedly accelerating the depletion of physiological reserves and compromising the patient’s ability to withstand the combined burden of sepsis and cancer. The inflammatory state reflected by hypoalbuminemia (33) closely overlaps with the pathophysiology of heart failure, including cytokine activation and oxidative stress. Patients with low GNRI already exhibit elevated systemic inflammation, which may further drive heart failure progression through a positive feedback loop (37), resulting in a sharp increase in mortality. Additionally, malnourished heart failure patients tend to have reduced tolerance to sepsis resuscitation measures such as fluid challenges and inotropic support, predisposing them to complications including fluid overload and renal dysfunction. 2. The stronger association between GNRI and mortality observed in patients with chronic pulmonary embolism may be attributed to the following interconnected mechanisms: (A) High energy expenditure and increased respiratory load: Chronic pulmonary embolism leads to pulmonary hypertension and right ventricular dysfunction (38, 39). The elevated work of breathing demands substantial energy, placing patients in a persistent hypermetabolic, high-energy-consumption state with significant pulmonary vascular resistance (40). When combined with low GNRI, respiratory muscles are prone to rapid fatigue, substantially raising the risk of respiratory failure and mortality compared to patients with adequate nutritional status. (B) Interaction between right heart failure and malnutrition: Chronic pulmonary embolism often progresses to right heart failure (cor pulmonale) (39). Similar to the mechanisms in the heart failure subgroup, malnutrition further compromises myocardial function, while right heart failure exacerbates gastrointestinal congestion and impairs nutrient absorption, creating a self-perpetuating cycle that accelerates clinical decline. (C) Hypoxia-induced metabolic dysregulation: Chronic hypoxia drives metabolic alterations (41), including anorexia and increased muscle catabolism. A poor nutritional status may exacerbate metabolic dysregulation, rendering patients more susceptible to multiorgan failure under acute infectious stress. Therefore, a low GNRI likely serves as a marker of critically depleted cardiopulmonary reserve (15, 42) and portends an extremely poor prognosis. 3. The differential prognostic value of GNRI for mortality in the subgroup receiving antitumor therapy (radiotherapy, chemotherapy, immunotherapy, or targeted therapy) compared to untreated patients may be explained by the following mechanisms: (A) Amplification of treatment toxicity: Nearly all current antitumor therapies carry side effects that can severely compromise nutritional intake and absorption. Patients with a low GNRI possess diminished physiological reserve, making them less able to tolerate such toxicities and more prone to severe treatment-related complications—such as serious infections or organ injury—thereby worsening outcomes in the setting of sepsis. (B) Critical threshold of immune function: Nutritional status serves as a cornerstone of immune competence (43). The efficacy of immunotherapy and certain chemotherapies depends on an adequate baseline immune response. Patients with a low GNRI may already be in a state of immune exhaustion, which could not only limit antitumor treatment effectiveness but also impair the ability to mount an adequate defense against sepsis. (C) Impaired anabolic capacity: Antitumor therapies are inherently catabolic, suppressing protein synthesis (44) and promoting muscle wasting. When coupled with a low GNRI—indicating already limited anabolic reserve—treatment can accelerate the progression of cachexia, leading to rapid functional decline. These factors underscore that, among patients who develop sepsis during active antitumor treatment, nutritional status is a critical determinant of their ability to survive this high-risk period.

In the MIMIC-IV database, GNRI showed no protective effect and even increased mortality in the subgroup with severe liver disease (p for interaction = 0.021). The underlying reasons are considered as follows: (A) Albumin is almost entirely synthesized by the liver; severe liver disease impairs synthetic function, and hypoalbuminemia primarily reflects hepatic reserve failure (20) rather than inadequate nutritional intake; (B) sepsis is characterized by systemic inflammatory response and capillary leakage, where hypoalbuminemia causes pathological weight gain due to generalized edema (5), resulting in falsely elevated GNRI values and underestimation of nutritional risk; (C) clinical observation indicates that in severe liver disease, complications of hepatic failure are more lethal and progress rapidly, with short-term mortality more directly dependent on management of acute complications rather than baseline nutritional status.

This study has several limitations. First, as a retrospective observational design, causal inference cannot be established, and residual confounding may remain despite multivariable adjustment. Second, GNRI depends on serum albumin and body weight, both of which can be acutely altered by critical illness, inflammation, fluid overload, and capillary leakage in sepsis. Although we adjusted for illness severity and inflammatory markers, acute-phase changes may still affect GNRI interpretation. Its strong predictive performance should not be interpreted purely as a nutritional indicator. Third, the Guangxi cohort is a single-center sample from a specialized cancer ICU, which may limit generalizability, although external validation in the MIMIC-IV database supports our findings. Fourth, some laboratory variables had missing data, which were handled by multiple imputation, but potential bias cannot be fully excluded. Fifth, detailed oncological information, including cancer stage, pathological type, and specific anti-tumor regimens, was not fully available. The MIMIC-IV database lacks specialized oncology data, and anti-tumor therapies in the cancer ICU cohort were administered prior to admission, with minimal influence on short-term mortality. Although we adjusted for prior anti-tumor therapy and metastatic status, future studies should incorporate comprehensive oncological stratification to further validate our findings. Sixth, data on the primary site of infection were not available in the current cohort. Although infection source affects sepsis severity and prognosis, all infectious triggers induce systemic inflammation and organ dysfunction. Multivariable analysis confirmed that GNRI was stably associated with mortality, suggesting that its prognostic value is independent of infection site. Future studies should incorporate detailed infection-site stratification. Finally, we focused on short-term mortality (28-day and 60-day), and longer-term outcomes were not assessed. Despite these limitations, GNRI demonstrated robust prognostic performance in both internal and external cohorts, supporting its clinical utility in this high-risk population.

## Conclusion

In adult patients with cancer and sepsis, a higher GNRI is associated with reduced all-cause mortality. As a nutritional risk stratification tool, GNRI demonstrates promising prognostic value for short- to medium-term prognosis in this high-risk population. The present findings support the use of GNRI to identify vulnerable subgroups, including patients with comorbid heart failure, chronic pulmonary embolism, or ongoing antitumor therapy, who may benefit from proactive, individualized early nutritional support to potentially improve outcomes. Additionally, the prognostic interpretation of GNRI in patients with severe liver disease requires caution.

## Data Availability

The raw data supporting the conclusions of this article will be made available by the authors, without undue reservation.

## Supplementary material

SUPPLEMENTARY FIGURE 1 Schoenfeld residual plots verifying the proportional hazards assumption of GNRI. **(A)** 28 day mortality model, Guangxi cohort; **(B)** 60 day mortality model, Guangxi cohort; **(C)** 28 day mortality model, MIMIC-IV cohort; **(D)** 60 day mortality model, MIMIC-IV cohort. Dotted gray line = zero-effect reference; cyan line = average hazard; red curve = time-dependent hazard trend; open circles = individual Schoenfeld residuals over follow-up days.
