# Supplementary figures and images for: The Geriatric Nutritional Risk Index and its association with all-cause mortality in cancer patients with sepsis: a dual-center retrospective cohort study
Source: Front Nutr. 2026 Jul 14;13:1795795. doi: 10.3389/fnut.2026.1795795 (PMC13407356; doi:10.3389/fnut.2026.1795795)

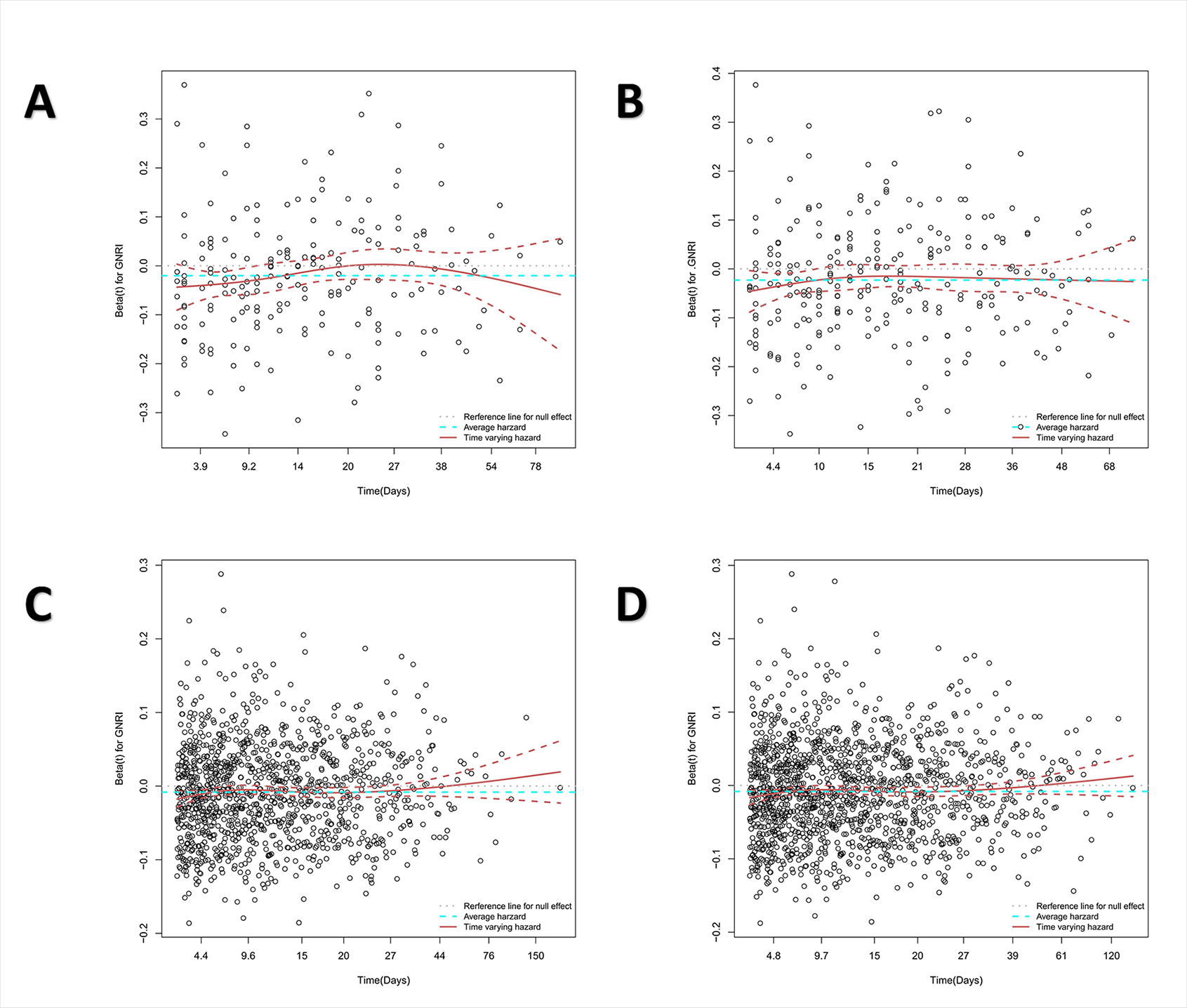

Supplement: SUPPLEMENTARY FIGURE 1 — Schoenfeld residual plots verifying the proportional hazards assumption of GNRI. (A) 28 day mortality model, Guangxi cohort; (B) 60 day mortality model, Guangxi cohort; (C) 28 day mortality model, MIMIC-IV cohort; (D) 60 day mortality model, MIMIC-IV cohort. Dotted gray line = zero-effect reference; cyan line = average hazard; red curve = time-dependent hazard trend; open circles = individual Schoenfeld residuals over follow-up days. [file Image_1.TIF]
