# Supplementary material for: The Geriatric Nutritional Risk Index and its association with all-cause mortality in cancer patients with sepsis: a dual-center retrospective cohort study
Source: Front Nutr. 2026 Jul 14;13:1795795. doi: 10.3389/fnut.2026.1795795 (PMC13407356; doi:10.3389/fnut.2026.1795795)
Supplement: Supplementary file 2 [file Table_1.DOCX]

**SUPPLEMENTARY TABLE 1**

**Baseline characteristics of the study populations(Guangxi Tumor Hospital and MIMIC-Ⅳ datebase)**

| Variables | **GNRI** (Guangxi Tumor Hospital) | | | | |  | **GNRI** (MIMIC-Ⅳ datebase) | | | | |
| --- | --- | --- | --- | --- | --- | --- | --- | --- | --- | --- | --- |
|  | Total  (n = 523) | T1  (n = 174) | T2  (n = 174) | T3  (n = 175) | *p* |  | Total  (n = 4447) | T1  (n = 1482) | T2  (n = 1482) | T3  (n = 1483) | *p* |
| Gender, n (%) |  |  |  |  | 0.264 |  |  |  |  |  | 0.488 |
| male | 323 (61.8) | 103 (59.2) | 116 (66.7) | 104 (59.4) |  |  | 2670 (60.0) | 887 (59.9) | 907 (61.2) | 876 (59.1) |  |
| female | 200 (38.2) | 71 (40.8) | 58 (33.3) | 71 (40.6) |  |  | 1777 (40.0) | 595 (40.1) | 575 (38.8) | 607 (40.9) |  |
| Age,year,  Mean ± SD | 59.3 ± 12.6 | 59.3 ± 11.7 | 58.4 ± 12.9 | 60.2 ± 13.2 | 0.398 |  | 71.0 ± 12.3 | 71.8 ± 13.0 | 71.9 ± 12.0 | 69.5 ± 11.8 | < 0.001 |
| Height,cm,  Mean ± SD | 162.4 ± 7.8 | 161.2 ± 8.1 | 163.3 ± 7.3 | 162.6 ± 8.0 | 0.046 |  | 169.2 ± 10.5 | 169.2 ± 10.3 | 169.4 ± 10.5 | 169.1 ± 10.7 | 0.754 |
| ABW,kg,  Mean ± SD | 57.5 ± 10.6 | 51.2 ± 9.4 | 57.7 ± 8.9 | 63.6 ± 9.6 | < 0.001 |  | 81.0 ± 21.7 | 67.6 ± 14.6 | 78.7 ± 16.2 | 96.6 ± 22.6 | < 0.001 |
| IBW,kg，  Mean ± SD | 58.9 ± 5.9 | 58.1 ± 6.0 | 59.6 ± 5.5 | 59.1 ± 6.1 | 0.064 |  | 63.8 ± 8.1 | 63.8 ± 8.0 | 64.0 ± 8.2 | 63.7 ± 8.3 | 0.643 |
| Heart Rate,  Mean ± SD | 113.1 ± 22.4 | 114.7 ± 23.1 | 111.4 ± 22.1 | 113.2 ± 22.0 | 0.393 |  | 86.9 ± 15.9 | 88.8 ± 17.2 | 86.3 ± 15.2 | 85.5 ± 15.2 | < 0.001 |
| MAP,mmHg,  Mean ± SD | 82.8 ± 18.9 | 78.5 ± 17.5 | 80.9 ± 16.9 | 88.9 ± 20.6 | < 0.001 |  | 75.9 ± 9.4 | 75.6 ± 9.6 | 75.8 ± 9.1 | 76.4 ± 9.5 | 0.052 |
| SOFA Score,  Mean ± SD | 9.5 ± 4.2 | 9.9 ± 4.1 | 9.2 ± 4.1 | 9.3 ± 4.4 | 0.254 |  | 3.8 ± 2.1 | 3.8 ± 2.1 | 3.8 ± 2.2 | 3.8 ± 2.1 | 0.826 |
| CAD, n (%) |  |  |  |  | 0.04 |  |  |  |  |  | 0.086 |
| no | 476 (91.0) | 166 (95.4) | 156 (89.7) | 154 (88) |  |  | 3648 (82.0) | 1230 (83) | 1189 (80.2) | 1229 (82.9) |  |
| yes | 47 ( 9.0) | 8 (4.6) | 18 (10.3) | 21 (12) |  |  | 799 (18.0) | 252 (17) | 293 (19.8) | 254 (17.1) |  |
| Stroke, n (%) |  |  |  |  | 0.426 |  |  |  |  |  | 0.697 |
| no | 448 (85.7) | 152 (87.4) | 151 (86.8) | 145 (82.9) |  |  | 3906 (87.8) | 1294 (87.3) | 1302 (87.9) | 1310 (88.3) |  |
| yes | 75 (14.3) | 22 (12.6) | 23 (13.2) | 30 (17.1) |  |  | 541 (12.2) | 188 (12.7) | 180 (12.1) | 173 (11.7) |  |
| SLD, n (%) |  |  |  |  | 0.57 |  |  |  |  |  | 0.292 |
| no | 366 (70.0) | 127 (73) | 119 (68.4) | 120 (68.6) |  |  | 4103 (92.3) | 1364 (92) | 1358 (91.6) | 1381 (93.1) |  |
| yes | 157 (30.0) | 47 (27) | 55 (31.6) | 55 (31.4) |  |  | 344 ( 7.7) | 118 (8) | 124 (8.4) | 102 (6.9) |  |
| MST, n (%) |  |  |  |  | 0.169 |  |  |  |  |  | < 0.001 |
| no | 225 (43.0) | 65 (37.4) | 78 (44.8) | 82 (46.9) |  |  | 3668 (82.5) | 1119 (75.5) | 1246 (84.1) | 1303 (87.9) |  |
| yes | 298 (57.0) | 109 (62.6) | 96 (55.2) | 93 (53.1) |  |  | 779 (17.5) | 363 (24.5) | 236 (15.9) | 180 (12.1) |  |
| MV, n (%) |  |  |  |  | 0.884 |  |  |  |  |  | 0.401 |
| no | 165 (31.5) | 57 (32.8) | 55 (31.6) | 53 (30.3) |  |  | 2215 (49.8) | 741 (50) | 755 (50.9) | 719 (48.5) |  |
| yes | 358 (68.5) | 117 (67.2) | 119 (68.4) | 122 (69.7) |  |  | 2232 (50.2) | 741 (50) | 727 (49.1) | 764 (51.5) |  |
| Vasopressin, n (%) |  |  |  |  | 0.089 |  |  |  |  |  | 0.192 |
| no | 134 (25.6) | 35 (20.1) | 46 (26.4) | 53 (30.3) |  |  | 1904 (42.8) | 652 (44) | 645 (43.5) | 607 (40.9) |  |
| yes | 389 (74.4) | 139 (79.9) | 128 (73.6) | 122 (69.7) |  |  | 2543 (57.2) | 830 (56) | 837 (56.5) | 876 (59.1) |  |
| RRT, n (%) |  |  |  |  | 0.01 |  |  |  |  |  | 0.259 |
| no | 441 (84.3) | 158 (90.8) | 138 (79.3) | 145 (82.9) |  |  | 3987 (89.7) | 1327 (89.5) | 1316 (88.8) | 1344 (90.6) |  |
| yes | 82 (15.7) | 16 (9.2) | 36 (20.7) | 30 (17.1) |  |  | 460 (10.3) | 155 (10.5) | 166 (11.2) | 139 (9.4) |  |
| Hormone, n (%) |  |  |  |  | 0.042 |  |  |  |  |  | < 0.001 |
| no | 444 (84.9) | 154 (88.5) | 151 (86.8) | 139 (79.4) |  |  | 3047 (68.5) | 962 (64.9) | 1015 (68.5) | 1070 (72.2) |  |
| yes | 79 (15.1) | 20 (11.5) | 23 (13.2) | 36 (20.6) |  |  | 1400 (31.5) | 520 (35.1) | 467 (31.5) | 413 (27.8) |  |
| WBC,10E9/L,  Median (IQR) | 10.4 (5.1, 17.6) | 9.4 (4.1, 16.3) | 10.8 (5.8, 19.5) | 10.7 (6.0, 17.4) | 0.292 |  | 11.9  (8.4, 15.9) | 12.2  (8.3, 16.7) | 11.7  (8.1, 15.6) | 11.9  (8.9, 15.5) | 0.097 |
| PLT,10e9/L,  Median (IQR) | 136.0 (62.0, 227.0) | 109.5 (50.8, 199.2) | 153.0 (69.8, 242.8) | 142.0 (78.5, 253.5) | 0.011 |  | 198.7 ± 113.6 | 215.5 ± 124.9 | 195.5 ± 116.7 | 185.3 ± 95.1 | < 0.001 |
| AG,mmol/L,  Mean ± SD | 12.5 ± 5.5 | 12.0 ± 5.9 | 12.2 ± 5.0 | 13.2 ± 5.5 | 0.103 |  | 14.6 ± 4.1 | 14.9 ± 4.2 | 14.6 ± 4.1 | 14.2 ± 3.9 | < 0.001 |
| P/F ratio,  Mean ± SD | 263.4 ± 123.6 | 260.8 ± 122.8 | 269.2 ± 130.4 | 259.9 ± 117.5 | 0.765 |  | 264.4 ± 126.5 | 261.0 ± 139.1 | 270.9 ± 131.7 | 261.2 ± 109.7 | 0.139 |
| LAC,mmol/L,  Median (IQR) | 2.6 (1.6, 4.2) | 2.9 (1.8, 5.2) | 2.5 (1.7, 3.9) | 2.3 (1.4, 3.7) | 0.006 |  | 2.0 (1.4, 3.0) | 2.0 (1.3, 3.2) | 2.0 (1.4, 2.9) | 2.0 (1.4, 2.8) | 0.524 |
| PT,sec,  Mean ± SD | 15.5 ± 6.4 | 16.7 ± 7.4 | 14.8 ± 6.2 | 14.8 ± 5.1 | 0.005 |  | 16.8 ± 8.5 | 17.7 ± 10.6 | 16.5 ± 7.1 | 16.3 ± 7.3 | < 0.001 |
| ALT,U/L, Median (IQR) | 21.0  (13.0, 47.0) | 19.5  (12.0, 38.8) | 24.0  (12.0, 55.0) | 22.0  (15.0, 53.0) | 0.121 |  | 29.0  (16.0, 72.0) | 29.5  (16.0, 65.0) | 28.5  (16.0, 85.0) | 29.0  (17.5, 77.9) | 0.241 |
| AST,U/L, Median (IQR) | 51.0  (30.0, 106.5) | 51.0  (33.0, 102.0) | 53.5  (30.0, 117.2) | 49.0  (29.0, 100.5) | 0.791 |  | 44.5 (26.0, 113.9) | 43.8 (26.0, 107.6) | 45.2 (26.0, 116.5) | 45.0 (27.0, 122.0) | 0.381 |
| 1. Day mortality,   n (%) |  |  |  |  | 0.112 |  |  |  |  |  | < 0.001 |
| no | 329 (62.9) | 99 (56.9) | 112 (64.4) | 118 (67.4) |  |  | 3304 (74.3) | 955 (64.4) | 1138 (76.8) | 1211 (81.7) |  |
| yes | 194 (37.1) | 75 (43.1) | 62 (35.6) | 57 (32.6) |  |  | 1143 (25.7) | 527 (35.6) | 344 (23.2) | 272 (18.3) |  |
| 1. Day mortality,   n (%) |  |  |  |  | 0.252 |  |  |  |  |  | < 0.001 |
| no | 270 (51.6) | 81 (46.6) | 93 (53.4) | 96 (54.9) |  |  | 3059 (68.8) | 838 (56.5) | 1051 (70.9) | 1170 (78.9) |  |
| yes | 253 (48.4) | 93 (53.4) | 81 (46.6) | 79 (45.1) |  |  | 1388 (31.2) | 644 (43.5) | 431 (29.1) | 313 (21.1) |  |

**Abbreviations**: ABW, Actual Body Weight ; IBW ,Ideal Body Weight ; MAP ,Mean Arterial Pressure ; SOFA,Sequential Organ Failure Assessment; CAD ,Coronary Artery Disease ; SLD ,Severe Liver Disease ; MST ,Metastatic Solid Tumor ; MV, Mechanical Ventilation ; RRT, Renal Replacement Therapy ; WBC, White Blood Cell ; PLT, platelet; AG,Anion gap ; P/F ratio, Oxygenation Index ; LAC,Lactic acid; PT, Prothrombin Time ; ALT,Alanine Aminotransferase; AST,Aspartate Aminotransferase; GNRI, Geriatric Nutritional Risk Index.
