# Supplementary material for: The Geriatric Nutritional Risk Index and its association with all-cause mortality in cancer patients with sepsis: a dual-center retrospective cohort study
Source: Front Nutr. 2026 Jul 14;13:1795795. doi: 10.3389/fnut.2026.1795795 (PMC13407356; doi:10.3389/fnut.2026.1795795)
Supplement: Supplementary file 3 [file Table_2.DOCX]

**Supplementary table 2: The proportional hazards model variable test for the relationship between GNRI and 28-day mortality rate model**

| **Variable** | **chisq** | **df** | **p.value** |
| --- | --- | --- | --- |
| GNRI | 1.481 | 1 | 0.224 |
| Gender | 0.86 | 1 | 0.354 |
| Age | 0.003 | 1 | 0.958 |
| Height | 0.325 | 1 | 0.569 |
| Hypertension | 0.407 | 1 | 0.524 |
| DM+CC | 0.887 | 1 | 0.346 |
| CAD | 1.375 | 1 | 0.241 |
| Heart Failure | 2.15 | 1 | 0.143 |
| Stroke | 0.023 | 1 | 0.879 |
| SLD | 0.055 | 1 | 0.815 |
| CLD | 2.098 | 1 | 0.148 |
| MST | 2.426 | 1 | 0.119 |
| APACHE-Ⅱ scores | 0.018 | 1 | 0.895 |
| SOFA scores | 1.885 | 1 | 0.17 |
| ATT(Chemo,TT,IO) | 3.387 | 1 | 0.066 |
| ATT(Radiotherapy) | 4.137 | 1 | 0.042 |
| Surgery | 2.911 | 1 | 0.088 |
| MV | 0.584 | 1 | 0.445 |
| Vasopressin | 0.012 | 1 | 0.913 |
| RRT | 5.585 | 1 | 0.018 |
| Hormone | 0.337 | 1 | 0.562 |
| Heart Rate | 0.008 | 1 | 0.929 |
| WBC | 0.631 | 1 | 0.427 |
| Lymphocytes | 0.135 | 1 | 0.713 |
| HGB | 2.586 | 1 | 0.108 |
| RDW | 0.004 | 1 | 0.951 |
| PLT | 0.11 | 1 | 0.74 |
| PCT | 1.914 | 1 | 0.166 |
| NT.pro-BNP | 0.088 | 1 | 0.766 |
| D-Dimer | 0.124 | 1 | 0.725 |
| PO2 | 0.005 | 1 | 0.942 |
| AG | 1.202 | 1 | 0.273 |
| P/F ratio | 0.399 | 1 | 0.527 |
| LAC | 0.132 | 1 | 0.716 |
| PT | 0.315 | 1 | 0.574 |
| APTT | 0.711 | 1 | 0.399 |
| FIB | 0.834 | 1 | 0.361 |
| ALT | 1.172 | 1 | 0.279 |
| CK | 0.186 | 1 | 0.666 |
| CK-MB | 0.282 | 1 | 0.596 |
| GLOBAL | 50.562 | 40 | 0.122 |

**Note**:GNRI and other variables all meet the proportional hazards (PH) assumption. This suggests that the main variables in the model have a stable impact on mortality risk within 28 days (the hazard ratio does not change over time). The global test indicates that the overall model meets the PH assumption, showing that the model is time-independent and robust for use.
