# Supplementary material for: The Geriatric Nutritional Risk Index and its association with all-cause mortality in cancer patients with sepsis: a dual-center retrospective cohort study
Source: Front Nutr. 2026 Jul 14;13:1795795. doi: 10.3389/fnut.2026.1795795 (PMC13407356; doi:10.3389/fnut.2026.1795795)
Supplement: Supplementary file 5 [file Table_4.DOCX]

**Supplementary table 4: The proportional hazards model variable test for the relationship between GNRI and 28-day mortality rate model**

| **Variable** | **chisq** | **df** | **p.value** |
| --- | --- | --- | --- |
| GNRI | 1.122 | 1 | 0.29 |
| gender | 0.518 | 1 | 0.472 |
| admission_age | 9.319 | 1 | 0.002 |
| Apsiii_score | 25.483 | 1 | 0 |
| sofa_score | 2.157 | 1 | 0.142 |
| myocardial_infarct | 0.687 | 1 | 0.407 |
| congestive_heart_failure | 2.144 | 1 | 0.143 |
| cerebrovascular_disease | 0.343 | 1 | 0.558 |
| chronic_pulmonary_disease | 2.163 | 1 | 0.141 |
| diabetes_with_cc | 1.509 | 1 | 0.219 |
| severe_liver_disease | 0.43 | 1 | 0.512 |
| ventilation | 1.772 | 1 | 0.183 |
| vasopressin | 4.633 | 1 | 0.031 |
| rrt | 2.552 | 1 | 0.11 |
| Hormone | 0.039 | 1 | 0.844 |
| wbc_mean | 0.073 | 1 | 0.787 |
| abs_lymphocytes_mean | 1.402 | 1 | 0.236 |
| hemoglobin_mean | 2.014 | 1 | 0.156 |
| platelets_mean | 0.901 | 1 | 0.343 |
| aniongap_mean | 40.79 | 1 | 0 |
| bun_mean | 7.366 | 1 | 0.007 |
| creatinine_mean | 1.005 | 1 | 0.316 |
| lactate_mean | 42.085 | 1 | 0 |
| P/F ratio_mean | 1.151 | 1 | 0.283 |
| pt_mean | 3.259 | 1 | 0.071 |
| aptt_mean | 0.174 | 1 | 0.676 |
| alt_mean | 10.531 | 1 | 0.001 |
| ast_mean | 14.25 | 1 | 0 |
| bilirubin_total_mean | 2.233 | 1 | 0.135 |
| GLOBAL | 97.224 | 29 | 0 |

**Note:** The GNRI satisfies the proportional-hazards (PH) assumption, implying its effect on 28-day mortality risk is constant over time. However, because some covariates are time-dependent, the global test shows that the overall model does not meet the PH assumption.
