# Supplementary material for: The Geriatric Nutritional Risk Index and its association with all-cause mortality in cancer patients with sepsis: a dual-center retrospective cohort study
Source: Front Nutr. 2026 Jul 14;13:1795795. doi: 10.3389/fnut.2026.1795795 (PMC13407356; doi:10.3389/fnut.2026.1795795)
Supplement: Supplementary file 6 [file Table_5.DOCX]

**Supplementary table 5: The proportional hazards model variable test for the relationship between GNRI and 60-day mortality rate model**

| **Variable** | **chisq** | **df** | **p.value** |
| --- | --- | --- | --- |
| GNRI | 1.742 | 1 | 0.187 |
| gender | 4.025 | 1 | 0.045 |
| admission_age | 11.294 | 1 | 0.001 |
| Apsiii_score | 23.235 | 1 | 0 |
| sofa_score | 4.768 | 1 | 0.029 |
| myocardial_infarct | 0 | 1 | 0.999 |
| congestive_heart_failure | 4.73 | 1 | 0.03 |
| cerebrovascular_disease | 1.079 | 1 | 0.299 |
| chronic_pulmonary_disease | 0.405 | 1 | 0.524 |
| diabetes_with_cc | 1.805 | 1 | 0.179 |
| severe_liver_disease | 3.508 | 1 | 0.061 |
| ventilation | 0.077 | 1 | 0.781 |
| vasopressin | 2.165 | 1 | 0.141 |
| rrt | 5.149 | 1 | 0.023 |
| Hormone | 3.773 | 1 | 0.052 |
| wbc_mean | 0.655 | 1 | 0.418 |
| abs_lymphocytes_mean | 1.12 | 1 | 0.29 |
| hemoglobin_mean | 0.146 | 1 | 0.702 |
| platelets_mean | 4.289 | 1 | 0.038 |
| aniongap_mean | 40.119 | 1 | 0 |
| bun_mean | 14.104 | 1 | 0 |
| creatinine_mean | 4.8 | 1 | 0.028 |
| lactate_mean | 27.281 | 1 | 0 |
| P/F ratio_mean | 1.372 | 1 | 0.241 |
| pt_mean | 5.19 | 1 | 0.023 |
| aptt_mean | 0.175 | 1 | 0.676 |
| alt_mean | 7.881 | 1 | 0.005 |
| ast_mean | 13.252 | 1 | 0 |
| bilirubin_total_mean | 6.54 | 1 | 0.011 |
| GLOBAL | 93.322 | 29 | 0 |

**Note:** The GNRI satisfies the proportional-hazards (PH) assumption, implying its effect on 60-day mortality risk is constant over time. However, because some covariates are time-dependent, the global test shows that the overall model does not meet the PH assumption.
