# Supplementary material for: The Geriatric Nutritional Risk Index and its association with all-cause mortality in cancer patients with sepsis: a dual-center retrospective cohort study
Source: Front Nutr. 2026 Jul 14;13:1795795. doi: 10.3389/fnut.2026.1795795 (PMC13407356; doi:10.3389/fnut.2026.1795795)
Supplement: Supplementary file 7 [file Table_6.DOCX]

**Supplementary table 6：Survival Landmark with a 7-Day Cutoff (MIMIC-Ⅳ)**

| Variable | n.total | n.event_% | crude.OR_95CI | crude.P_value |
| --- | --- | --- | --- | --- |
| Time below the landmark(7-day) | Time below the landmark | Time below the landmark | Time below the landmark | Time below the landmark |
| GNRI31 | 1482 | 181 (12.2) | 1(Ref) |  |
| GNRI32 | 1482 | 115 (7.8) | 0.641 (0.507~0.809) | <0.001 |
| GNRI33 | 1483 | 94 (6.3) | 0.541 (0.422~0.694) | <0.001 |
| Trend.test | 4447 | 390 (8.8) | 0.725 (0.639~0.821) | <0.001 |
| Time over the landmark(7-day) | Time over the landmark | Time over the landmark | Time over the landmark | Time over the landmark |
| GNRI31 | 1113 | 346 (31.1) | 1(Ref) |  |
| GNRI32 | 1040 | 229 (22) | 0.827 (0.7~0.977) | 0.0259 |
| GNRI33 | 911 | 178 (19.5) | 0.814 (0.679~0.975) | 0.0257 |
| Trend.test | 3064 | 753 (24.6) | 0.893 (0.816~0.978) | 0.0146 |
