# Supplementary material for: The Geriatric Nutritional Risk Index and its association with all-cause mortality in cancer patients with sepsis: a dual-center retrospective cohort study
Source: Front Nutr. 2026 Jul 14;13:1795795. doi: 10.3389/fnut.2026.1795795 (PMC13407356; doi:10.3389/fnut.2026.1795795)
Supplement: Supplementary file 8 [file Table_7.DOCX]

**Supplementary table 7：Survival Landmark with a 14-Day Cutoff (MIMIC-Ⅳ)**

| Variable | n.total | n.event_% | crude.OR_95CI | crude.P_value |
| --- | --- | --- | --- | --- |
| Time below the landmark(14-day) | Time below the landmark | Time below the landmark | Time below the landmark | Time below the landmark |
| GNRI31 | 1482 | 340 (22.9) | 1(Ref) |  |
| GNRI32 | 1482 | 240 (16.2) | 0.772 (0.654~0.911) | 0.0021 |
| GNRI33 | 1483 | 182 (12.3) | 0.642 (0.536~0.769) | <0.001 |
| Trend.test | 4447 | 762 (17.1) | 0.798 (0.73~0.873) | <0.001 |
| Time over the landmark(14-day) | Time over the landmark | Time over the landmark | Time over the landmark | Time over the landmark |
| GNRI31 | 651 | 187 (28.7) | 1(Ref) |  |
| GNRI32 | 489 | 104 (21.3) | 0.729 (0.573~0.927) | 0.0099 |
| GNRI33 | 408 | 90 (22.1) | 0.863 (0.671~1.11) | 0.2512 |
| Trend.test | 1548 | 381 (24.6) | 0.903 (0.794~1.026) | 0.1184 |
