# Supplementary material for: The Geriatric Nutritional Risk Index and its association with all-cause mortality in cancer patients with sepsis: a dual-center retrospective cohort study
Source: Front Nutr. 2026 Jul 14;13:1795795. doi: 10.3389/fnut.2026.1795795 (PMC13407356; doi:10.3389/fnut.2026.1795795)
Supplement: Supplementary file 9 [file Table_8.DOCX]

**Supplementary table 8. Internal cross-validation results for the predictive model**

| Cross-validation fold | Original C‑index | Cross‑validated C‑index | Standard error (SE) | Optimism‑corrected C‑index |
| --- | --- | --- | --- | --- |
| Fold 1 | 0.732 | 0.728 | 0.018 | 0.726 |
| Fold 2 | 0.735 | 0.729 | 0.017 | 0.727 |

Note: The optimism‑corrected C‑index was calculated as: Original C‑index − (Original C‑index − Cross‑validated C‑index). The small decreases after cross‑validation indicate minimal overfitting.
